# Thermostability and pH differentiation in *Geobacillus* hydrolases: a comparative perspective

**DOI:** 10.1093/jimb/kuag027

**Published:** 2026-10-08

**Authors:** Elifnur Akbiyik, Ayse Varol, Zehra Betul Tekgul, Berna Genc, Ahmet Adiguzel

**Affiliations:** Department of Molecular Biology and Genetics, Faculty of Science, Ataturk University, Erzurum, Turkiye; Department of Molecular Biology and Genetics, Faculty of Science, Ataturk University, Erzurum, Turkiye; Department of Molecular Biology and Genetics, Faculty of Science, Ataturk University, Erzurum, Turkiye; Department of Food Engineering, Faculty of Engineering and Life Sciences, Gumushane University, Gumushane, Turkiye; Department of Molecular Biology and Genetics, Faculty of Science, Ataturk University, Erzurum, Turkiye

**Keywords:** *Geobacillus*, thermostable hydrolases, thermophilic adaptation, comparative enzymology, catalytic maxima (*T*_max_ and pH_max_)

## Abstract

Thermostable enzymes are central to high-temperature industrial processes because they combine catalytic efficiency with structural resilience under demanding conditions. Among thermophilic bacteria, *Geobacillus* has emerged as a prominent source of industrially relevant enzymes, particularly hydrolases such as proteases, lipases, and carbohydrate-active enzymes. Although many individual enzymes have been characterized in detail, comparative synthesis across catalytic classes within the genus remains limited. In this review, we integrate biochemical data and structural evidence to explore whether thermostability in *Geobacillus* reflects isolated enzyme-specific traits or recurring functional patterns across enzyme classes. Reported *T*_max_ and pH_max_ values from purified and partially purified hydrolases were systematically compiled to assess class-level tendencies. Temperature optima cluster within a relatively narrow thermophilic range across enzyme groups, supporting high-temperature activity as a common feature within the genus. In contrast, pH optima display clearer functional differentiation, with proteases and lipases consistently shifted toward alkaline values relative to carbohydrate-active hydrolases. Recurrent stabilizing elements, including reinforced hydrophobic cores, expanded electrostatic interaction networks, and a balanced rigidity–flexibility profile, are observed across multiple enzyme classes, indicating shared structural trends rather than independent enzyme-specific variation. By situating catalytic optima within a comparative framework and relating them to shared structural determinants, this review provides a comparative interpretation of patterns observed across *Geobacillus*-derived enzymes, linking structure, catalysis, and functional adaptation.

**One-sentence summary** Thermostable enzymes from *Geobacillus* thrive at similar high temperatures but diverge in pH behavior, revealing simple patterns that can guide smarter use in biotechnology.

## Graphical Abstract

**Graphical Abstract:**
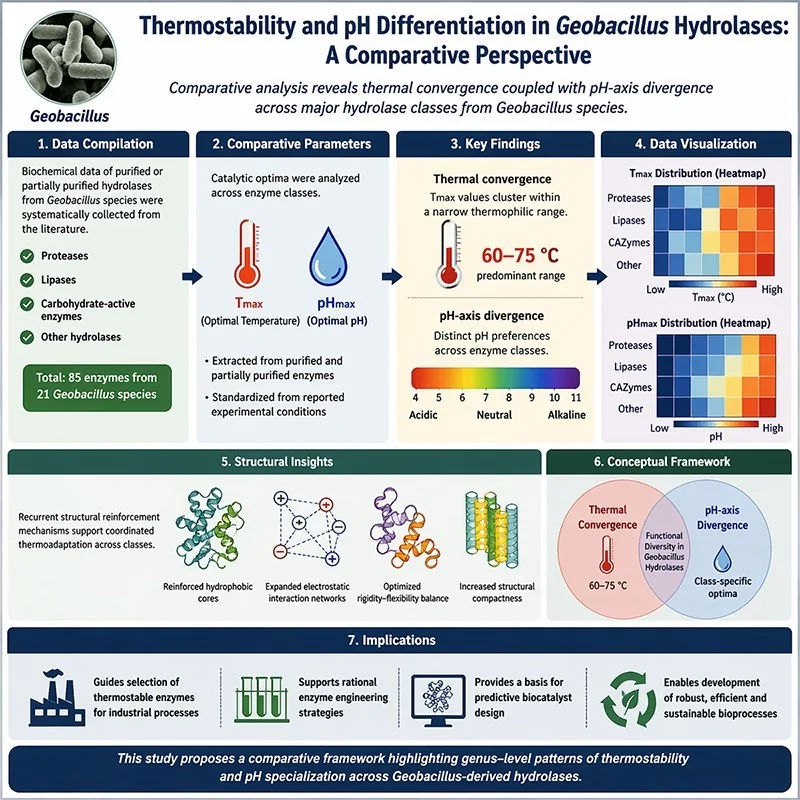

## Highlights

- This review proposes a comparative framework highlighting genus-level patterns of thermostability in *Geobacillus*, moving beyond enzyme-specific descriptions.
- Comparative analysis reveals thermal convergence coupled with pH-axis divergence across major hydrolase classes.
- The findings indicate that pH specialization, rather than temperature adaptation, is a key driver of functional diversification in thermophilic enzymes.
- Recurrent structural reinforcement mechanisms support the concept of coordinated thermoadaptation across *Geobacillus*-derived enzymes.
- This integrative framework provides a basis for predictive and application-oriented selection of thermostable enzymes in industrial and biotechnological systems.

## Introduction

Thermophilic enzymes occupy a central role in modern industrial biotechnology, as their ability to maintain catalytic activity and structural integrity under high temperatures and chemically demanding conditions has made them indispensable across sectors ranging from food processing and detergents to bioenergy and biorefinery platforms (Rigoldi et al., 2018). Compared to mesophilic enzymes, thermophilic enzymes generally possess advantages such as longer working stability, lower risk of contamination during processing, and improved reaction kinetics at high temperatures, leading to a continuously increasing interest in identifying and characterizing potent biocatalysts derived from thermophilic microorganisms (Sharma et al., 2019).

Among thermophilic bacteria, the genus *Geobacillus* has become recognized as an important reservoir of enzymes with industrial relevance (Lebre et al., 2018). Originally classified among thermophilic members of *Bacillus*, the genus was subsequently established as a distinct taxonomic group comprising Gram-positive, endospore-forming bacteria adapted to elevated temperatures, with growth reported at temperatures up to approximately 80 °C. Members of the genus are commonly isolated from geothermal sites, composting systems, oil reservoirs, and other thermally influenced environments (Brumm et al., 2016; Najar & Thakur, 2020; Sari et al., 2018). The ecological distribution of *Geobacillus* reflects its continuous exposure to heat stress, a selective pressure that shapes its genomic composition and proteomic stability. Consequently, a variety of enzymes isolated from *Geobacillus*, including proteases, lipases, amylases, xylanases, cellulases, and other glycoside hydrolases, have been shown to be thermostable, generally alkaliphilic, and adaptable to harsh industrial environments (Aliyu et al., 2016).

Over the past two decades, significant efforts have been devoted to the cloning, expression, purification, and biochemical characterization of *Geobacillus* enzymes. These studies have provided detailed information on protein engineering strategies, including optimal temperature (*T*_max_), optimal pH (pHmax), substrate specificity, and structural characteristics (rather than emphasizing methodological optimization alone) (Aliyu et al., 2016; Brumm et al., 2016). These studies collectively confirm that *Geobacillus* is a reliable source of thermophilic biocatalysts. However, most current research is enzyme-centric, focusing on single strains or isolated catalytic classes. Therefore, broader comparative studies of different enzyme types, structural determinants, and adaptation patterns within this genus remain limited. Due to the fragmentation of related research, it remains inconclusive whether the thermostability of *Geobacillus* represents only a group of independently characterized enzymes or reflects recurring functional and structural patterns across multiple catalytic categories (Najar & Thakur, 2020). Addressing this gap requires a comparative evaluation of whether catalytic optima and structural stabilization features recur across different *Geobacillus* enzyme classes rather than representing isolated enzyme-specific observations (Vieille & Zeikus, 2001).

In this context, the present review aims to comparatively examine catalytic optima (Tmax and pHmax) and associated structural features across major hydrolase classes in *Geobacillus*. By synthesizing reported catalytic optima (*T*_max_ and pH_max_) and integrating these observations with recurrent structural features, we identify and discuss shared patterns across enzyme groups without attributing them to a unified or coordinated evolutionary mechanism. This perspective provides a comparative and integrative interpretation linking structure, catalysis, and functional adaptation in *Geobacillus*, while maintaining a data-driven and non-speculative framework.

## Taxonomic and genomic background

The genus *Geobacillus* has undergone substantial taxonomic revision since its establishment, with several species historically assigned to the genus subsequently transferred to other genera. According to the current LPSN taxonomy, *Geobacillus* comprises 12 species with validly published and correct names. This number should not be equated with the substantially larger number of genome assemblies available in NCBI, because multiple genome assemblies may correspond to different strains of the same species. The currently accepted *Geobacillus* species are summarized in Table 1. Several taxa historically assigned to *Geobacillus* have subsequently been reclassified or are currently regarded as synonyms; therefore, historical species names are retained in this review when referring to enzymes reported under those names in the original literature. The current taxonomic classification of *Geobacillus* species is summarized in Table 2. While the genus *Bacillus* comprises a phylogenetically diverse group of Gram-positive, endospore-forming bacteria, comparative genomic analyses revealed that thermophilic lineages form a distinct evolutionary cluster warranting separation at the genus level. Advances in whole-genome sequencing, including average nucleotide identity and in silico DNA–DNA hybridization analyses, enabled high-resolution phylogenetic delineation and supported the establishment of *Geobacillus* as a separate genus (Pogrebnyakov et al., 2017). These genomic tools clarified species boundaries and provided insights into evolutionary divergence among thermophilic Firmicutes. The phylogenetic relationships among representative *Geobacillus* species are illustrated in Figure 1, highlighting the clustering of thermophilic lineages within a distinct evolutionary group. The genus *Geobacillus* comprises multiple thermophilic species with diverse physiological and ecological characteristics.

**Figure 1:**
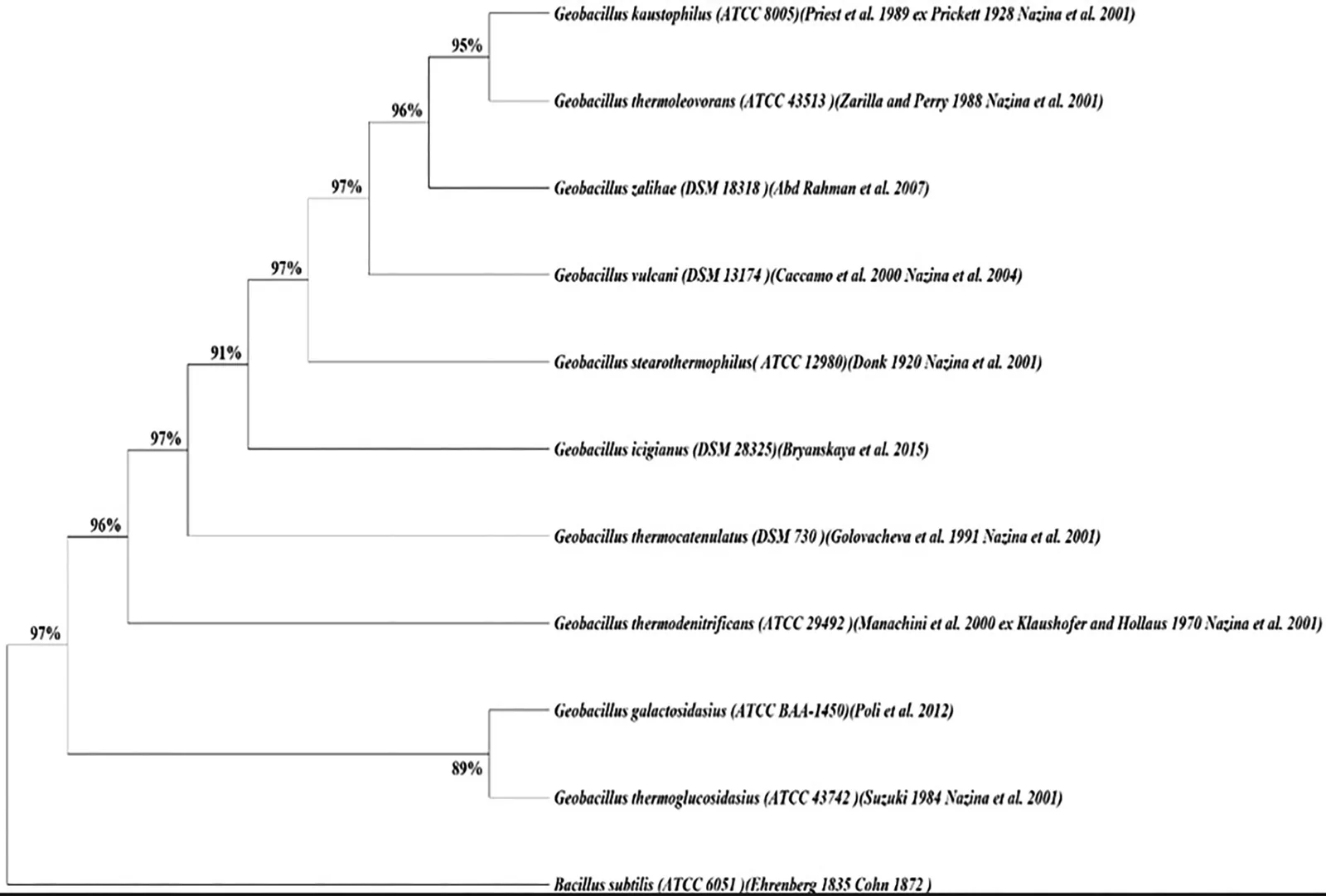
Neighbor-joining phylogenetic tree showing the relationships among representative *Geobacillus* species, with *Bacillus subtilis* included as the outgroup. Bootstrap support values are shown at the corresponding nodes. Evolutionary analyses were conducted using MEGA10 (Bryanskaya et al., 2015; Cohn, 1872; Donk, 1920; Golovacheva et al., 1975; Manachini et al., 2000; Nazina et al., 2001, 2004; Poli et al., 2011; Priest et al., 1988; Rahman et al., 2007; Zarilla & Perry, 1987).

**Table 1:** Currently recognized *Geobacillus* species with validly published and correct names according to LPSN.

Name	Category	Taxonomic status	URL
*Geobacillus kaustophilus*	Species	Correct name	https://lpsn.dsmz.de/species/geobacillus-kaustophilus
*Geobacillus icigianus*	Species	Correct name	https://lpsn.dsmz.de/species/geobacillus-icigianus
*Geobacillus jurassicus*	Species	Correct name	https://lpsn.dsmz.de/species/geobacillus-jurassicus
*Geobacillus lituanicus*	Species	Correct name	https://lpsn.dsmz.de/species/geobacillus-lituanicus
*Geobacillus stearothermophilus*	Species	Correct name	https://lpsn.dsmz.de/species/geobacillus-stearothermophilus
*Geobacillus proteiniphilus*	Species	Correct name	https://lpsn.dsmz.de/species/geobacillus-proteiniphilus
*Geobacillus subterraneus*	Species	Correct name	https://lpsn.dsmz.de/species/geobacillus-subterraneus
*Geobacillus thermocatenulatus*	Species	Correct name	https://lpsn.dsmz.de/species/geobacillus-thermocatenulatus
*Geobacillus thermodenitrificans*	Species	Correct name	https://lpsn.dsmz.de/species/geobacillus-thermodenitrificans
*Geobacillus thermoleovorans*	Species	Correct name	https://lpsn.dsmz.de/species/geobacillus-thermoleovorans
*Geobacillus uzenensis*	Species	Correct name	https://lpsn.dsmz.de/species/geobacillus-uzenensis
*Geobacillus vulcani*	Species	Correct name	https://lpsn.dsmz.de/species/geobacillus-vulcani

**Table 2:** Current taxonomic classification.

Domain	Bacteria
Phylum	*Bacillota*
Class	*Bacilli*
Order	*Caryophanales*
Family	*Anoxybacillaceae*
Genus	*Geobacillus*

Members of the genus *Geobacillus* are Gram-positive, rod-shaped, endospore-forming thermophiles, exhibiting optimal growth temperatures typically between 45 and 80 °C. They display metabolic versatility, including obligate aerobic, denitrifying, and facultative anaerobic capabilities (Hussein et al., 2015). Optimal growth generally occurs within a pH range of 6.0–8.5 (Novik et al., 2019). Beyond physiological traits, genomic and transcriptomic studies have identified heat-responsive proteostasis systems in *Geobacillus*, including the major molecular chaperones DnaK, DnaJ, GroEL, GroES, and GrpE, as well as ATP-dependent Clp proteins (Wang et al., 2019, 2020). In *G. stearothermophilus* B5, heat-shock-associated genes were upregulated at 70 °C relative to 60 °C, with fold increases of 2.1–5.3; specifically, ClpX, Hsp33, DnaK, DnaJ, ClpB, GrpE, and ClpP showed 2.1-, 2.3-, 2.7-, 2.8-, 2.8-, 4.5-, and 5.3-fold increases, respectively (Wang et al., 2019). These systems contribute to protein folding, refolding, and quality control during thermal stress, thereby supporting cellular proteostasis at elevated temperatures (Wang et al., 2019, 2020).

Recent phylogenomic analyses have placed the genus *Geobacillus* within the family *Anoxybacillaceae* rather than the traditionally used Bacillaceae, based on genome-scale phylogeny and conserved molecular signatures (Patel et al., 2024). The current taxonomic classification is presented in Table 2.

### Representative species and physiological diversity

The genus *Geobacillus* comprises multiple thermophilic species exhibiting diverse physiological and metabolic characteristics. While detailed species-level descriptions are available in taxonomic studies, a concise overview is presented here to illustrate species diversity without detracting from the enzyme-focused scope of this review.

Representative species demonstrate variations in growth temperature, pH tolerance, and ecological adaptation. For example, *G. stearothermophilus* is among the most extensively studied species due to its strong enzyme production capacity and industrial relevance, with reported growth between 30 and 75 °C and near-neutral pH conditions (Burgess et al., 2017; Chang et al., 2021). *Geobacillus thermodenitrificans* exhibits metabolic flexibility, including denitrification and the ability to utilize long-chain hydrocarbons, reflecting adaptation to diverse ecological niches (Balan et al., 2012; Panosyan et al., 2018).

*Geobacillus kaustophilus*, originally isolated from deep-sea sediments, is characterized by growth up to ~74 °C, tolerance to moderate salinity, and broad substrate utilization capacity (Suzuki et al., 2012; Takami, Nishi, et al., 2004). Similarly, *G. thermocatenulatus* demonstrates adaptability to a wide temperature range (37–80 °C) and variable environmental conditions, including salinity tolerance (Dinsdale et al., 2011; Jo et al., 2021).

In addition, species such as *G. galactosidasius* exhibit neutrophilic growth behavior (optimum pH ~7.2) and the ability to utilize a wide range of carbon sources, indicating metabolic versatility (Najar & Thakur, 2020; Poli et al., 2011; Sari et al., 2018). Other members of the genus, including *G. thermoglucosidasius, G. thermoleovorans, G. vulcani, G. zalihae*, and *G. icigianus*, collectively highlight the ecological breadth and functional diversity of *Geobacillus*, particularly in high-temperature and hydrocarbon-rich environments (Najar et al., 2020; Pogrebnyakov et al., 2017). Some of these species have been explored for applications such as biomass degradation, bioremediation, and high-temperature fermentation (Cripps et al., 2009; Dinsdale et al., 2011).

Overall, these representative species illustrate the metabolic diversity, thermal robustness, and ecological adaptability that characterize the genus *Geobacillus*. Representative physiological characteristics of selected *Geobacillus* species are summarized in Table 3.

**Table 3:** Selected physiological characteristics of representative *Geobacillus* species

Species	Growth temperature (°C)	Growth pH	Key characteristics	Reference
*G. galactosidasius*	50–75 (opt. 70)	6–8 (opt. 7.2)	Broad carbon source utilization; aerobic	(Najar & Thakur, 2020; Poli et al., 2011; Sari et al., 2018)
*G. kaustophilus*	up to 74 (opt. 60)	6.2–7.5	Broad substrate utilization; salt tolerance (>3% NaCl); nitrate reduction	(Suzuki et al., 2012; Takami, Takaki, et al., 2004)
*G. stearothermophilus*	30–75	6.0–7.5	Widely studied; strong enzyme production potential	(Burgess et al., 2017; Chang et al., 2021)
*G. thermocatenulatus*	37–80 (opt. 60)	5–9 (opt. 7)	Salt tolerance (5% NaCl); adaptable growth conditions	(Dinsdale et al., 2011; Jo et al., 2021)
*G. thermodenitrificans*	45–73 (opt. 65)	~7	Denitrification; hydrocarbon utilization (C15–C36)	(Balan et al., 2012; Panosyan et al., 2018)
*G. thermoglucosidasius*	55–75	Neutral–alkaline	Strong carbohydrate metabolism; fermentation applications	(Cripps et al., 2009; Najar & Thakur, 2020)
*G. thermoleovorans*	55–75	Neutral–alkaline	Hydrocarbon degradation; geothermal adaptation	(Dinsdale et al., 2011; Najar & Thakur, 2020)
*G. vulcani*	~Thermophilic	~Neutral	Adapted to volcanic/geothermal environments; potential enzyme source	(Najar & Thakur, 2020; Nazina et al., 2004)
*G. zalihae*	Thermophilic	Neutral–alkaline	Lignocellulolytic enzyme production (xylanases)	(Najar & Thakur, 2020)
*G. icigianus*	Thermophilic	~Neutral	Underexplored; potential novel thermostable enzymes	(Najar & Thakur, 2020)

## Thermophilic enzyme producers: the role of *Geobacillus*

Microorganisms constitute the primary source of industrial enzymes due to their rapid growth, scalable cultivation, and genetic tractability. Among them, thermophilic bacteria have attracted particular attention because their enzymes retain catalytic activity under elevated temperatures and harsh processing conditions. Thermophilic organisms experience strong selective pressure for protein stability, and their enzymes commonly exhibit structural adaptations that limit thermally induced unfolding while maintaining the local flexibility required for catalysis (Sterpone & Melchionna, 2012; Vieille & Zeikus, 2001).

Beyond their role as sources of thermostable enzymes, *Geobacillus* species are increasingly being investigated as microbial platforms for high-temperature bioprocessing. Their metabolic versatility enables the utilization of diverse carbon sources, while advances in genome-scale metabolic modeling and genetic engineering have expanded their potential as thermophilic cell factories. For example, a genome-scale metabolic model developed for the fast-growing thermophile *Geobacillus* sp. LC300 demonstrated the feasibility of predicting growth and metabolic fluxes under thermophilic conditions and provided a framework for rational strain engineering (Ljungqvist & Gustavsson, 2022). More recently, metabolic engineering of *G. thermoglucosidasius* enabled the production of 151.1 g/L L-lactic acid and 153.1 g/L D-lactic acid at 60 °C, illustrating the potential of thermophilic *Geobacillus* strains for high-temperature production of industrial chemicals (Liu et al., 2024). These developments extend the biotechnological relevance of *Geobacillus* beyond enzyme discovery and support its emerging role as a host for thermophilic biomanufacturing.

## Ancillary manifestations of genus-level thermoadaptation in *Geobacillus*: industrial relevance and catalytic adaptation

Hydrolases constitute the most extensively studied and industrially exploited enzyme class produced by *Geobacillus* species (Table 4). The dataset compiled in Table 4 forms the basis for the comparative analyses presented in Section “Comparative Catalytic Landscape and Statistical Assessment of *T*_max_ and pH_max_”. These enzymes catalyze the hydrolytic cleavage of diverse chemical bonds, including peptide, ester, and glycosidic linkages, under elevated temperature conditions that are often incompatible with mesophilic biocatalysts. Despite extensive application-oriented research, reliable market-size estimates specifically attributable to *Geobacillus*-derived enzymes are not publicly available, making it difficult to quantify their commercial contribution independently from the broader industrial enzyme market. Nevertheless, established applications have been documented; notably, thermolysin produced by *G. stearothermophilus* has been evaluated for use as a processing aid in the manufacture of enzyme-modified dairy ingredients, yeast extracts, cooked food products, and protein hydrolysates derived from several animal and plant sources. While hydrolases represent the most extensively characterized functional outputs of thermoadaptation in *Geobacillus*, genus-level thermal robustness is not confined to catalytic proteins alone. A growing body of evidence indicates that ancillary traits such as antimicrobial peptide production, exopolysaccharide synthesis, and biofilm-associated functionalities also exhibit enhanced stability and functionality under elevated temperatures. These features may therefore be viewed as associated manifestations of thermophilic adaptation rather than direct outcomes of a unified genus-level mechanism.

**Table 4:** Representative thermostable hydrolases derived from Geobacillus spp., including reported temperature optima (*T*_max_) and pH optima (pH_max_) values compiled from the literature.

	Enzyme	Microorganism	Temperature of highest activity (°C)	pH of highest activity	Reference
1	α-Amylase	*Geobacillus kaustophilus* TSCCA02	60	9.0	(Phonlamai et al., 2024)
2	α-Amylase	*Geobacillus thermodenitrificans* DSM-465	70	8.0	(Balan et al., 2012; (Daas et al., 2017)
3	α-Amylase	*Geobacillus sp*. GS33	60	7–8	(Burhanoğlu et al., 2020)
4	α-Amylase	*Geobacillus sp*.	80	6.0	(Sudan et al., 2018)
5	α-Amylase	*Geobacillus stearothermophilus* SR74	65	7.0	(Gandhi et al., 2015)
6	α-Amylase	*Geobacillus stearothermophilus*	70	7.0	(Al-Qodah, 2006)
7	α-Amylase	*Geobacillus stearothermophilus*	70	7.0	(Fincan & Enez, 2014)
8	α-Amylase	*Geobacillus sp*. IIPTN	80	5.0	(Dheeran et al., 2010)
9	Maltogenic Amylase	*Geobacillus sp*. SK70	55	7.0	(Sulong, 2017)
10	Pullulanase	*Geobacillus stearothermophilus* ADM-11	70	7.0	(Abbas Bukhari et al., 2024)
11	Amylopullulanase	*Geobacillus thermoleovorans* NP1	50	6.0	(Arabacı & Arıkan, 2019)
12	Xylanase	*Geobacillus thermoleovorans* AKNT10	100	6.0	(Kumar et al., 2024)
13	Xylanase	*Geobacillus sp*.	75	7.0	(Bibra et al., 2018)
14	Xylanase	*Geobacillus vulcani* GS90	55	8.0	(Algan et al., 2021)
15	Xylanase	*Geobacillus galactosidasius* BS61	60	7.0	(Sari et al., 2018)
16	V171S lipase	*Geobacillus sp. 42*	70 °C	8.0	(Johan et al., 2025)
17	Lipase	*Geobacillus kaustophilus*	50 °C	8.0	(Tülek et al., 2025)
18	Lipase	*Geobacillus* sp. ID17	50 °C	11.0	(Salas-Bruggink et al., 2024)
19	Lipase	*Geobacillus thermoleovorans*	76 °C	9.5	(Moharana et al., 2019)
20	Lipase	*Geobacillus kaustophilus* DSM 7263 ^T^	50 °C	8.0	(Özdemir et al., 2021)
21	T1 lipase	*Geobacillus zalihae*	70 °C	9.0	(Maiangwa et al., 2021)
22	GDSL-type lipase	*Geobacillus thermocatenulatus*	60 °C	9.0	(Jo et al., 2021)
23	Monoacylglycerol lipase	*Geobacillus* sp.	70 °C	9.0	(Liu et al., 2023)
24	Thermoalkaliphilic lipase	*Geobacillus stearothermophilus* FMR12	70 °C	10.0	(Abol-Fotouh et al., 2021)
25	Protease	*Geobacillus thermoglucosidasius* SKF4	70 °C	10.0	(Allison et al., 2024)
26	Serine protease	*Geobacillus stearothermophilus*	50 °C	8.5	(Zebrowska et al., 2022)
27	Serine protease	*Geobacillus* sp. GS53	55 °C	8.0	(Baykara et al., 2021)
*28*	*Metalloprotease*	*Geobacillus thermoleovorans HBB208*	70°C	8.0	(Karaman & Metin 2025)
					
29	Protease	*Geobacillus* SBS-4S	60 °C	9.0	(Ahmad et al., 2020)
30	Serine protease	*Geobacillus stearothermophilus* B-1172	90 °C	9.0	(Iqbal et al., 2015)
31	Serine protease AprX	*Geobacillus thermoleovorans* ARTRW1	55 °C	8.0	(Oztug et al., 2022)

*Note*. The dataset comprises enzymes from multiple hydrolase classes and serves as the basis for the comparative analyses presented in Section “Comparative Catalytic Landscape and Statistical Assessment of *T*_max_ and pH_max_”.

The hydrolase repertoire of *Geobacillus* spp. includes proteases, lipases, amylases, esterases, xylanases, cellulases, and α-glucosidases, many of which demonstrate broad pH tolerance and remarkable resistance to surfactants, oxidants, and organic solvents. These characteristics underpin their widespread application in food processing, detergents, bioenergy production, biomass conversion, and pharmaceutical manufacturing. A comparative overview of thermostable hydrolases derived from *Geobacillus* spp., including their catalytic properties and industrial applications, is presented in Table 5.

**Table 5:** Comparative overview of thermostable hydrolases from *Geobacillus* spp. with reported catalytic properties and industrial applications.

Enzyme (strain)	Optimum/reported catalytic conditions (*T*_max_, pH_max_)	Example application	References
α-Amylase (*Geobacillus* sp. GS33, DS3, *G. icigianus*)	Active at 50–70 °C; pH 6–8; Ca²⁺-activated; long-term thermal stability	Starch hydrolysis; bakery; porous starch production	(Burhanoğlu et al., 2020; Kurniawan et al., 2024; Soy et al., 2021; Widiana et al., 2022)
Xylanase (*G. stearothermophilus* GF16; *G. vulcani* GS90)	Active at 30–90 °C; pH 3–9; high thermostability	Biomass-to-xylose conversion; juice clarification	(Algan et al., 2021; Carbonaro et al., 2024)
Cellulase (CMCase) (*Geobacillus* sp. KP43)	Optimum ~70 °C; stable under prolonged incubation; Fe²⁺-stimulated	High-temperature cellulose degradation	(Khadka et al., 2022)
Serine protease (*Geobacillus* sp. GS53)	Optimum 55 °C; pH 8; stable at elevated temperatures and in surfactants	Detergent; leather; food processing	(Baykara et al., 2021; Wang et al., 2025)
Lipase (*G. zalihae; G. stearothermophilus* FMR12)	Thermoalkaline activity (≈60–70 °C; pH 9–10); solvent and detergent tolerance	Biodiesel; detergent formulations	(Abol-Fotouh et al., 2021; Maiangwa et al., 2021)
Esterase (EstA) (*G. thermoleovorans* YN)	Optimum 60–65 °C; retains activity after thermal incubation	Pharmaceutical intermediates; fine chemicals	(Soliman et al., 2007)
α-Glucosidase (*Geobacillus* spp.)	Active at 60–70 °C; neutral to slightly alkaline pH	Oligosaccharide synthesis; saccharification	(Zhang et al., 2019)

### Proteases

Proteases are hydrolytic enzymes that cleave peptide bonds, converting proteins into smaller peptides and free amino acids. They are classified according to their catalytic mechanism, including serine, metalloproteases, cysteine, and aspartic proteases. From an industrial standpoint, proteases represent the dominant segment of the global enzyme market, accounting for approximately 60% of total commercial enzyme production. Their applications extend across food processing, pharmaceuticals, detergents, and leather treatment industries (Li et al., 2012).

Proteases synthesized by *Geobacillus* species are particularly notable for their stability under extreme physicochemical conditions, including elevated temperatures, alkaline pH, chemical denaturants, organic solvents, and surfactants. These enzymes are predominantly extracellular and maintain catalytic activity over wide temperature and pH ranges, making them highly suitable for harsh industrial environments. Alkaline proteases are widely employed in detergent formulations for the removal of protein-based stains (Baykara et al., 2021). *Geobacillus stearothermophilus* has been identified as a prolific source of industrially relevant proteases, frequently isolated from soil and geothermal habitats. An alkaline serine protease from *G. stearothermophilus* F1, isolated from composted oil palm branches, was initially cloned and heterologously expressed in *Escherichia coli*. However, expression in *E. coli* resulted in inclusion body formation and improper folding, reducing active enzyme yield. To overcome these limitations, *Pichia pastoris* was employed as an alternative host for secretory expression, improving protein folding and catalytic recovery (Ma et al., 2016).

Similarly, the alkaline protease gene from *G. stearothermophilus* B-1172 was cloned and heterologously expressed in *E. coli*, followed by purification and biochemical characterization of the recombinant enzyme (Iqbal et al., 2015). Several studies have reported the production, purification, and characterization of a thermostable alkaline protease from *Geobacillus stearothermophilus*, demonstrating its applicability in bio-detergent formulations (Baykara et al., 2021; Chang et al., 2021). Protease production increased substantially under optimized culture conditions, reinforcing the feasibility of industrial-scale enzyme synthesis.

A thermostable serine protease isolated from *Geobacillus toebii* LBT exhibited exceptional stability in the presence of haloalkanes, organic solvents, and SDS (sodium dodecyl sulfate). Such resilience under chemically aggressive environments further supports the suitability of *Geobacillus*-derived proteases for demanding industrial processes (Najar & Thakur, 2020). In another study, *Geobacillus* sp. YMTC 1049, isolated from a hot spring in Rehai, Tengchong (Yunnan Province, China), was reported to produce a thermostable extracellular serine protease (RH-1). The enzyme displayed maximal activity at 85 °C and pH 7.5 and retained stability at 65 °C for up to 10 hr, underscoring the intrinsic thermal robustness characteristic of proteases derived from thermophilic *Geobacillus* strains (Zhu et al., 2007).

Taken together, proteases derived from *Geobacillus* exhibit a consistent convergence toward thermophilic activity maxima coupled with a moderate but reproducible alkaline shift. While individual proteases differ in catalytic mechanism and structural family, their shared stability under high temperature and alkaline conditions suggests that protease robustness can be interpreted as reflecting broader adaptive tendencies rather than strictly isolated enzyme optimization. This pattern is consistent with the presence of recurring structural and functional trends within the genus, without implying a coordinated evolutionary framework.

### Lipases

Lipases are members of the lipolytic enzyme family that catalyze the hydrolysis of triacylglycerols and other water-insoluble esters at the lipid–water interface. In addition to hydrolytic activity, lipases are capable of catalyzing esterification, alcoholysis, and interesterification reactions which makes them versatile biocatalysts in both aqueous and non-aqueous systems. Although lipases share catalytic similarities with esterases, they differ in substrate specificity. Unlike esterases, which preferentially act on soluble short-chain esters, lipases primarily hydrolyze insoluble lipid substrates in aqueous environments, exhibiting a preference for medium- and long-chain fatty acids (Sürmeli, Tekedar and Şanlı-Mohamed, 2024).

Lipases are produced by plants, animals, fungi, yeasts, and bacteria. Among these, microbial lipases, particularly bacterial lipases, are of greater industrial relevance because they can be produced rapidly and at scale, are amenable to genetic and protein engineering, and can catalyze both hydrolytic and synthetic reactions in aqueous and non-aqueous systems (Viveke et al., 2022). Many microbial lipases possess conserved catalytic machinery and exhibit industrially relevant properties such as broad substrate specificity and activity or stability in organic solvents; in lipases containing a mobile lid domain, interfacial activation represents an important determinant of catalytic behavior (Khan et al., 2017; Vivek et al., 2022). Lipases are widely used in the detergent, food, dairy, pulp, and pharmaceutical industries. In particular, microbial lipases have attracted considerable interest due to their versatile properties, which enable their application across diverse industrial sectors, including pulp and paper, pharmaceuticals, food processing, biodiesel production, cosmetics, and detergents (Vivek et al., 2022).

A thermostable lipase was purified and characterized from *Geobacillus thermodenitrificans* IBRL-nra. The enzyme exhibited maximum activity at pH 7.0 and remained stable for up to 16 hr at this pH when incubated at 65 °C. In a separate study, high-level expression of a thermostable lipase from *Geobacillus* sp. strain T1 was reported. Following optimization, one of the recombinant lipases demonstrated high specific activity in wet cells, with an optimum pH of 9 and an optimum temperature of 65 °C. This study highlighted rapid cloning and overexpression strategies that enabled high enzymatic yields, underscoring the enzyme’s potential for industrial applications. Similarly, a thermostable lipase from *G. thermoleovorans* Toshki was cloned and overexpressed in *E. coli*. The recombinant expression system exhibited approximately 4.5-fold higher lipase activity than that produced by the native *G. thermoleovorans* Toshki strain. Notably, the lipase was stable at 60 °C and pH 8 and retained about 30% of its residual activity after 1 hr of incubation at 100 °C, demonstrating remarkable thermal stability (Bustos-Baena et al., 2025; Qin et al., 2024).

Collectively, these findings underscore the catalytic robustness and industrial relevance of *Geobacillus*-derived lipases, particularly for high-temperature and alkaline processing environments. In comparison to proteases, lipases from *Geobacillus* demonstrate a more pronounced displacement toward alkaline pH optima while maintaining thermophilic temperature preferences. Structurally, many lipases contain a mobile lid domain that covers the catalytic site in the closed conformation and undergoes conformational rearrangement upon interaction with a hydrophobic interface, thereby exposing the active site and facilitating substrate access. Lid architecture and dynamics can influence interfacial activation, substrate specificity, catalytic efficiency, and enzyme stability (Khan et al., 2017). In thermostable *Geobacillus* lipases, structural analyses have further highlighted the contribution of hydrophobic interactions and conformational features surrounding the lid region to catalytic performance and thermal stability (Moharana et al., 2019). Although the recurrent occurrence of thermoalkaliphilic lipases across *Geobacillus* strains supports pH specialization as a class-dependent functional trend, the extent to which lid dynamics or other structural features directly determine this alkaline shift remains to be established. The structural organization of the catalytic site and the functional role of the lid region are illustrated in Figure 2.

**Figure 2:**
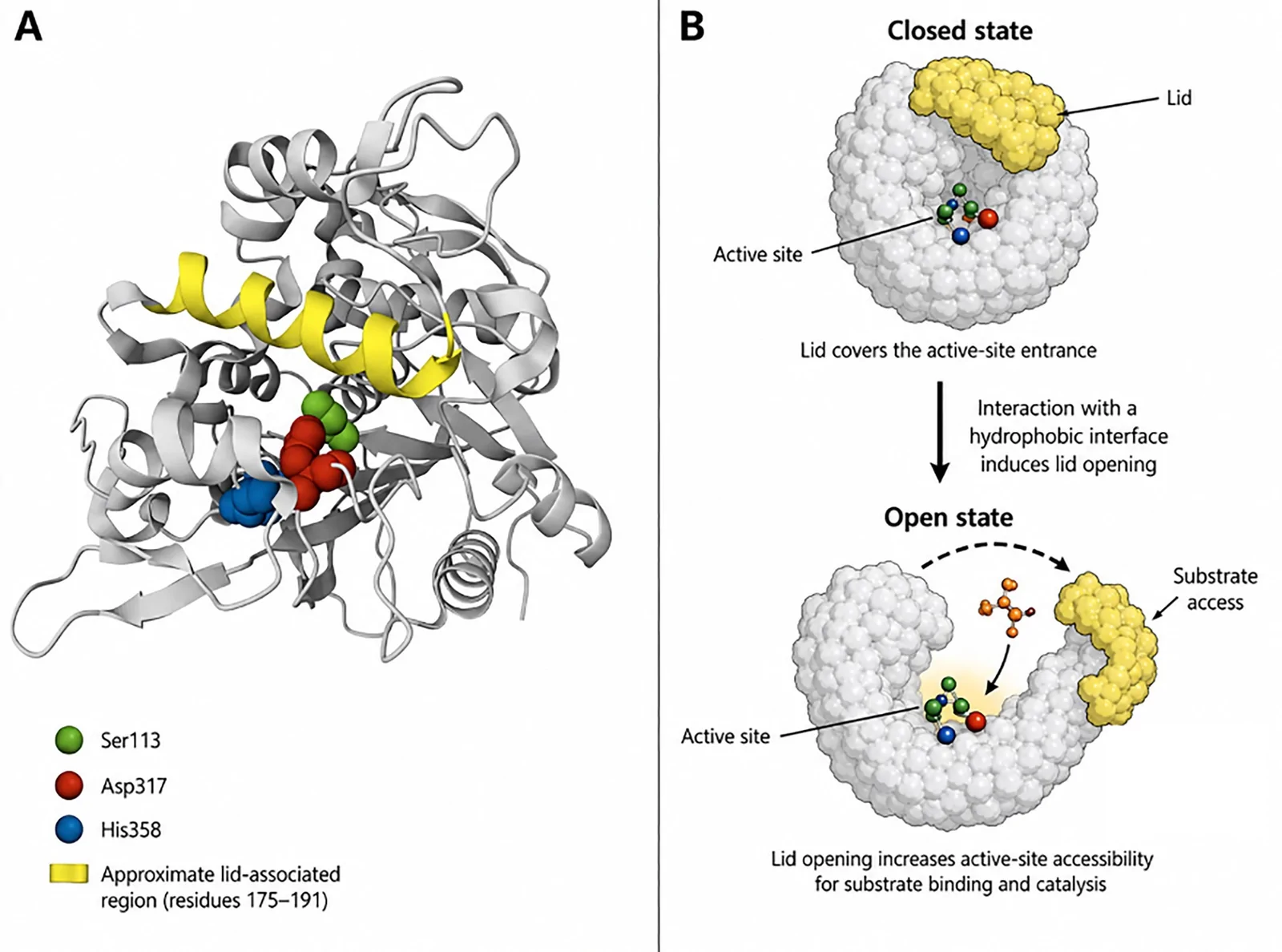
Structural organization and functional role of the lid-associated region in a thermostable Geobacillus thermoleovorans lipase. (A) Crystal structure of *G. thermoleovorans* lipase rendered from PDB entry 6A12 (Moharana et al., 2019). The catalytic triad residues Ser113, Asp317, and His358 are shown, respectively. Residues 175–191 are highlighted as an approximate lid-associated region to provide spatial orientation relative to the catalytic site; this highlighting is not intended to define the complete residue-level boundaries of the lid domain. (B) Conceptual schematic of lid-mediated interfacial activation. In the closed state, the lid restricts access to the active-site entrance. Interaction with a hydrophobic interface promotes conformational rearrangement of the lid, increasing active-site accessibility and facilitating substrate binding and catalysis. The open state in panel B is schematic and does not represent an experimentally determined open conformation of PDB 6A12.

### Esterases

Esterases belong to the lipolytic hydrolase family and primarily catalyze the hydrolysis of short-chain, water-soluble ester substrates (typically <10 carbon atoms), releasing alcohols and fatty acids. These enzymes are involved in various reactions such as esterification, transesterification, and interesterification. Structurally, most esterases belong to the α/β-hydrolase fold family and possess a conserved catalytic triad composed of Ser–Asp–His residues (Samoylova et al., 2018).

Esterase enzymes are widely distributed in plants, animals, and microorganisms. The most commercially useful sources of esterase are microorganisms, particularly bacteria, yeasts, and fungi, due to their ease of cultivation, genetic manipulability, and scalable production. They find widespread applications in the pharmaceutical, cosmetic, detergent, food, textile, and paper industries (Sürmeli et al., 2024). For instance, EstA isolated from *Geobacillus thermoleovorans* YN exhibits optimal activity at 60–65 °C and retains its functionality after 1 hr of incubation at this temperature. Additionally, *Geobacillus* sp. ZH1 shows negligible loss of activity after 3 hr at 60°C and maintains more than 40% of its activity after 3 hr at 90 °C (Zhu et al., 2012). These findings underscore the marked thermal stability of *Geobacillus*-derived esterases. By comparison, the thermolabile mesophilic HSL-family esterase E40 retained only 17% of its activity after 1 hr at 30 °C and was completely inactivated after 6 min at 40 °C (Li et al., 2015), illustrating the substantially greater thermal persistence of the *Geobacillus* esterases described above.

### α–Amylases

α-Amylase is an enzyme that cleaves α,1–4 glycosidic bonds within starch polymers such as amylose and amylopectin, acting as an endo-amylase that releases glucose, maltose, and dextrins. These enzymes are widely distributed in nature and can be produced by a broad range of microorganisms, particularly bacteria. α-Amylases occur in bacterial, archaeal, and eukaryotic lineages. Bacterial α-amylases exhibit particularly broad physicochemical and catalytic diversity, including variation in temperature and pH optima, substrate specificity, and hydrolysis-product profiles (Mehta & Satyanarayana, 2016). Phylogenetic analyses further indicate substantial evolutionary divergence among α-amylases from bacterial, archaeal, and eukaryotic origins (Yan & Wu, 2016). The optimum pH of bacterial α-amylases ranges widely from 1.0 to 11.5, making enzymes from moderately thermophilic bacteria particularly suitable for industrial applications at elevated temperatures (Al-Amri et al., 2021).

Physiologically, most α-amylases possess two aspartate and one glutamate residues that play critical roles in catalysis. α-amylases play a key role in starch digestion in the human body. Beyond their physiological function, α-amylases represent one of the most commercially significant enzyme groups due to their catalytic versatility and robustness under process conditions (Khaswal et al., 2022). Currently, chemical hydrolysis of starch at the industrial scale has largely been replaced by amylase-catalyzed processes. α-Amylases are of considerable importance in the textile, fermentation, food, paper, and related industries and represent approximately 25% of the global enzyme market (Ahmed et al., 2021).

Amylases are widely used in the fruit, vegetable, and juice industries to facilitate juicing, clarification, and quality improvement. Cloudy juices are clarified through the combined action of amylases with pectinases and cellulases. In the textile industry, α-amylase is extensively applied for desizing, where starch used during weaving is removed prior to dyeing to improve fabric smoothness and quality. *Geobacillus thermoleovorans* has been reported as a potent producer of α-amylase. Under optimized cultivation conditions, the enzyme exhibited high specific activity at 70 °C (Uma Maheswar Rao & Satyanarayana, 2007).

The broad pH tolerance and thermal resilience of bacterial α-amylases further enhance their suitability for high-temperature starch processing. *Geobacillus sp*. IIPTN, isolated from hot springs in the Himalayan region of Uttarakhand, was reported to be a potent producer of thermostable α-amylase, exhibiting high specific activity when cultivated at pH 6.5 and approximately 60 °C. Consistent with the general characteristics of the genus *Geobacillus*, which includes thermophilic bacteria adapted to high temperatures and known for producing thermostable enzymes, *Geobacillus* sp. GS33 has been reported to produce a thermostable α-amylase exhibiting maximum activity at pH 7–8 and 60 °C (Khaswal et al., 2022).

Importantly, *Geobacillus*-derived amylases have also entered established food-processing applications. Maltogenic α-amylase derived from *G. stearothermophilus* is used in wheat-bread production, particularly in commercial enzyme preparations applied as antistaling agents to retard crumb firming. Commercial preparations containing *G. stearothermophilus* maltogenic α-amylase have been identified in baking applications, and residual activity of this enzyme has been detected after bread baking (Rebholz et al., 2021; Reichenberger et al., 2019).

### Xylanases

Xylanases, formally classified as endo-1,4-β-xylanases, catalyze the hydrolysis of β-1,4-xylosidic bonds of xylan within the hemicellulosic backbone. Xylan is a structurally complex heteropolysaccharide composed of a β-1,4-linked xylose backbone substituted with side groups such as 4-O-methyl-d-glucuronosyl and α-arabinofuranosyl residues. These enzymes constitute a key class of biocatalysts involved in the degradation of hemicellulosic polysaccharides in biomass (Phuyal et al., 2023).

Many industrial processes operate under elevated temperatures and variable pH conditions, highlighting the suitability of thermostable xylanases derived from thermophilic microorganisms. Xylan-processing applications rely heavily on xylanases because of their environmentally favorable properties and their potential to reduce chemical usage and processing costs (Phuyal et al., 2023). They are widely applied in the food, textile, feed, and pulp and paper industries. In the paper industry, xylanases enhance the efficiency of bleaching agents such as hydrogen peroxide, ozone, and chlorine, thereby reducing chemical load. In juice and wine production, they facilitate clarification processes and improve product quality.

Several thermostable xylanases from *Geobacillus* have been characterized, with representative enzymes displaying activity across thermophilic temperature ranges. A thermo-alkali-stable xylanase from *G. thermoleovorans* has been investigated for lignocellulosic processing and xylooligosaccharide production (Verma & Satyanarayana, 2012). More recently, an ultra-thermostable xylanase from *G. thermoleovorans* AKNT10 was characterized for wheat-bran saccharification (Kumar *et al*., 2024). In addition, the xylanase from *G. galactosidasius* BS61 exhibited maximal activity at approximately 60 °C and was characterized using both conventional and molecular approaches (Sari et al., 2018).

Collectively, these findings emphasize that *Geobacillus*-derived xylanases combine high thermal tolerance with alkaline adaptability, making them particularly attractive for lignocellulosic biomass conversion and environmentally sustainable industrial processing.

### Cellulases

Cellulose, the most abundant biopolymer on Earth, is hydrolyzed by a multienzyme complex collectively referred to as cellulases, which include endoglucanases, exoglucanases, and β-glucosidases. Since thermostable enzymes retain their activity for longer periods at elevated temperatures, they enable more efficient cellulose degradation, thereby lowering overall production costs. Consequently, there is a growing demand for highly stable, active, and substrate-specific cellulases that can be produced in a cost-effective manner (Ajeje et al., 2021). In this context, members of the genus *Geobacillus* have emerged as promising sources of thermostable cellulases due to their ability to thrive at high temperatures and produce industrially robust enzymes. These enzymes play a critical role in various industrial sectors such as food processing, detergents, textiles, pulp, and paper industries, and are also extensively exploited in bioethanol production (Ajeje et al., 2021). Thermostable cellulases are particularly advantageous in lignocellulosic biomass conversion because elevated process temperatures can reduce medium viscosity, improve feedstock solubility and substrate diffusion, and lower the risk of microbial contamination (Ajeje et al., 2021).

A thermostable cellulase produced by *Geobacillus* strain WSUCF1 retained 89% of its initial CMCase activity after incubation at 70 °C for 24 hr and exhibited optimal activity at 70 °C and pH 5, demonstrating prolonged catalytic stability under elevated-temperature conditions (Rastogi et al., 2010).

### α-Glucosidases

α-Glucosidases are hydrolytic enzymes belonging to glycoside hydrolase (GH) families and participate in the hydrolysis of a range of glycosidic substrates, including disaccharides, oligosaccharides, polysaccharides, and aromatic glycosides. Thermophilic α-glucosidases have been characterized from *Geobacillus* strains, and several enzymes have also been successfully cloned and heterologously expressed for improved production (Zhang et al., 2019).

In addition to thermal tolerance, several reported enzymes display broad substrate specificity and stability across a wide pH range, characteristics that enhance their applicability in starch processing, oligosaccharide synthesis, and biofuel-related saccharification steps. Due to their thermostability, broad substrate specificity, and industrial robustness, α-glucosidases derived from *Geobacillus* spp. represent promising biocatalysts for biotechnological and industrial applications (Zhang et al., 2019).

Unlike proteases and lipases, carbohydrate-active hydrolases from *Geobacillus* consistently cluster around near-neutral pH optima despite operating within a thermophilic temperature window. This constrained pH distribution may reflect mechanistic requirements of glycosidic bond cleavage, where precise protonation states of catalytic residues can limit alkaliphilic drift. The coexistence of conserved thermophilic temperature optima with restricted pH flexibility indicates that thermophilic activity provides a common baseline across enzyme classes, while catalytic chemistry imposes class-specific constraints on pH adaptation.

## Additional functional traits in *Geobacillus*

Although *Geobacillus* species are primarily recognized for their thermostable hydrolases, a growing number of studies suggest that their functional repertoire extends beyond classical biocatalysis. These additional traits include antimicrobial peptide production, exopolysaccharide (EPS) synthesis, and metabolic capabilities relevant to environmental and energy-related processes. However, compared to hydrolase-focused research, these areas remain less systematically explored and should be interpreted cautiously within the context of enzyme-centered studies.

Thermophilic members of the genera *Geobacillus* and *Parageobacillus* have been investigated as potential sources of bacteriocins and bacteriocin-like inhibitory substances (BLISes). Among these, geobacillin I and II remain the best-characterized lantibiotic bacteriocins, with defined genetic and physicochemical properties (Yu et al., 2023). In addition to these classical compounds, several isolates have been reported to produce BLISes exhibiting inhibitory activity against Gram-positive bacteria across broad temperature ranges (Zebrowska et al., 2022). Despite these promising observations, current evidence remains limited and largely strain-specific, with challenges related to activity spectrum, stability, and scalability (Zebrowska et al., 2022).

Thermophilic adaptation also enables certain *Geobacillus* species to produce structurally stable exopolysaccharides. Beyond their structural and protective roles, some EPS preparations have demonstrated *in vitro* antioxidant activity, particularly through DPPH-based assays (Aytar et al., 2024; Ismail et al., 2025; Wang et al., 2025). However, these findings are primarily based on chemical assays, and further work is required to better understand the relationship between EPS structure and biological function under physiological conditions.

In addition to bioactive compound production, several *Geobacillus* species exhibit metabolic versatility that supports their use in environmental and industrial applications. For example, some strains are capable of degrading hydrocarbons, phenolic compounds, and dyes, as well as participating in heavy metal biosorption processes (Najar & Thakur, 2020). Furthermore, *Geobacillus* species contribute to biofuel-related systems through lignocellulosic biomass conversion and biodiesel-associated reactions, and their integration into anaerobic digestion systems has been associated with improved biogas yields (Khaswal et al., 2022).

Biofilm formation represents another important characteristic of *Geobacillus*, particularly in thermophilic and industrial environments. While biofilms may enhance survival under stress conditions, they also present challenges in food-processing systems, where they can lead to contamination, equipment fouling, and reduced process efficiency (Kilic, 2020).

Overall, these observations indicate that *Geobacillus* exhibits a broader functional diversity beyond hydrolase production. However, since most findings remain strain-specific and relatively limited in scope, they should be interpreted cautiously and not generalized at the genus level.

## Structural adaptation mechanisms conferring thermostability

Thermostability in enzymes derived from thermophilic *Geobacillus* species does not arise from a single structural feature but rather from a combination of molecular adaptations operating at sequence, structural, and dynamic levels. These adaptations collectively preserve conformational integrity, minimize thermal unfolding, and sustain catalytic efficiency under elevated temperatures. The following subsections synthesize the primary determinants underlying thermostability in *Geobacillus* enzymes and present them within a comparative conceptual framework (Aytar et al., 2024; Hussein et al., 2015; Novik et al., 2019). A comparative summary of structural determinants associated with thermostability in *Geobacillus*-derived enzymes is presented in Table 6.

**Table 6:** Qualitative synthesis of recurrent structural and catalytic features reported in *Geobacillus*-derived hydrolases.

Structural feature	Proteases	Lipases	CAZymes	Genus-level tendency	References
Hydrophobic core enrichment	Frequently reported	Frequently reported	Reported	Recurrent across enzyme classes	(Arya et al., 2024; Wang et al., 2025)
Salt bridge density	Frequently reported	Reported	Reported	Common stabilization feature across multiple enzymes	(Modarres et al., 2016; Sürmeli et al., 2024)
Disulfide stabilization	Reported (context-dependent)	Reported	Less commonly described	Variable contribution depending on enzyme class	(Zhang et al., 2021)
Alkaline pH shift	Moderate alkaline tendency	Strong alkaline tendency	Predominantly near-neutral	Class-dependent variation in pH preference	(Hasan et al., 2010; Saraçoğlu et al., 2013)
Rigidity–flexibility tuning	Active-site flexibility discussed	Lid-domain dynamics emphasized	Acid-base catalytic constraint	Shared structural tendency related to catalytic function	(Sterpone & Melchionna, 2012; Wang et al., 2025)

### Sequence-level determinants of thermoadaptation

Thermostability in *Geobacillus* enzymes is strongly influenced by amino acid composition and its impact on protein folding landscapes. Comparative analyses between thermophilic and mesophilic homologs consistently reveal enrichment in hydrophobic and aromatic residues, including isoleucine and proline, which enhance packing density within the hydrophobic core and reduce conformational entropy at elevated temperatures (Modarres et al., 2016; Nezhad et al., 2022).

In parallel, increased frequencies of charged residues such as lysine and glutamic acid promote formation of surface-exposed ionic interactions and salt bridges, contributing to electrostatic stabilization (Arya et al., 2024). Importantly, thermostable proteins also exhibit reduced proportions of thermolabile residues susceptible to deamidation or conformational instability. These sequence biases do not merely alter primary structure; they reshape the folding energy landscape. Thermophilic proteins tend to favor deeper global energy minima, thereby reducing the probability of unfolding transitions under thermal stress.

### Structural reinforcement networks

Beyond sequence composition, thermostability depends on cooperative structural interactions. Disulfide bonds and salt bridges function not as isolated stabilizing elements but as components of interconnected stabilization networks.

Disulfide bonds, when strategically positioned, constrain conformational flexibility and reinforce tertiary structure. Rational introduction of additional disulfide bridges into *Geobacillus* α-amylase increased optimal temperature and extended half-life, illustrating how covalent crosslinks enhance thermal tolerance (Zhang et al., 2021). However, improper placement may destabilize folding intermediates, emphasizing the necessity of structural context (Wang et al., 2025). Salt bridges represent another dominant stabilizing feature. Thermostable proteins typically exhibit a higher density of ionic interactions compared to mesophilic counterparts (Modarres et al., 2016; Nezhad et al., 2022). These interactions function as “structural clamps” that stabilize surface regions while permitting core rigidity (Sterpone & Melchionna, 2012). In *Geobacillus* esterases, salt bridges located within cap domains significantly enhanced thermal stability (Sürmeli et al., 2024). Arginine residues, due to their guanidinium groups, frequently contribute to multi-point ionic networking (Veltman et al., 1996). Importantly, thermostability correlates not simply with the number of ionic bonds but with the formation of cooperative electrostatic networks that balance desolvation penalties and entropic constraints (Berezovsky et al., 2017; Robinson-Rechavi & Godzik, 2005).

### Rigidity–flexibility balance and catalytic function

Thermostability in *Geobacillus* proteases appears to depend on maintaining a stable global fold while preserving localized conformational mobility required for catalysis. Recent molecular-dynamics analysis of a thermophilic serine protease from *G. stearothermophilus* showed that the enzyme maintained overall structural integrity at elevated temperatures while retaining region-specific fluctuations, supporting a balance between global rigidity and local flexibility (Jamal et al., 2025). Such dynamic partitioning may allow thermophilic proteases to resist thermal unfolding without excessively restricting the conformational changes required for substrate recognition and catalytic turnover.

### An integrative thermostability model for *Geobacillus* enzymes

The molecular determinants described above operate in concert rather than independently. Thermostability in *Geobacillus* enzymes can be described as a multilayered combination of interacting factors, including: (i) sequence-level hydrophobic enrichment and charged residue optimization, (ii) dense hydrophobic core packing, (iii) cooperative ionic and disulfide bonding networks, (iv) controlled conformational flexibility at catalytic sites, and (v) genomic variability that may facilitate adaptive responses. Collectively, these features contribute to enzymes capable of sustained catalytic performance under thermophilic and often alkaline industrial environments. Importantly, the structural mechanisms outlined here directly underpin the functional properties discussed in Section “Ancillary manifestations of genus-level thermoadaptation in *Geobacillus*: Industrial Relevance and Catalytic Adaptation”. The alkaline stability of proteases, solvent tolerance of lipases, and extended half-life of α-amylases can be interpreted as reflecting different expressions of shared stabilization features.

## Comparative catalytic landscape and statistical assessment of *T*_max_ and Ph_max_

Although numerous studies have characterized individual hydrolases derived from *Geobacillus* species, the reported catalytic parameters are typically discussed on an enzyme-by-enzyme basis. In most cases, authors report the temperature and pH at which maximum activity is observed, yet these *T*_max_ and pH_max_ values are not systematically synthesized across functional classes (Najm et al., 2022; Wang et al., 2025). To address this fragmentation, we compiled reported temperature of maximum activity (hereafter *T*_max_) and pH of maximum activity (pH_max_) values from peer-reviewed studies focusing on purified or partially purified *Geobacillus* hydrolases. As summarized in Figure 3, the thermophilic phenotype of *Geobacillus* enzymes is characterized by thermal convergence coupled with pH-axis diversification.

**Figure 3:**
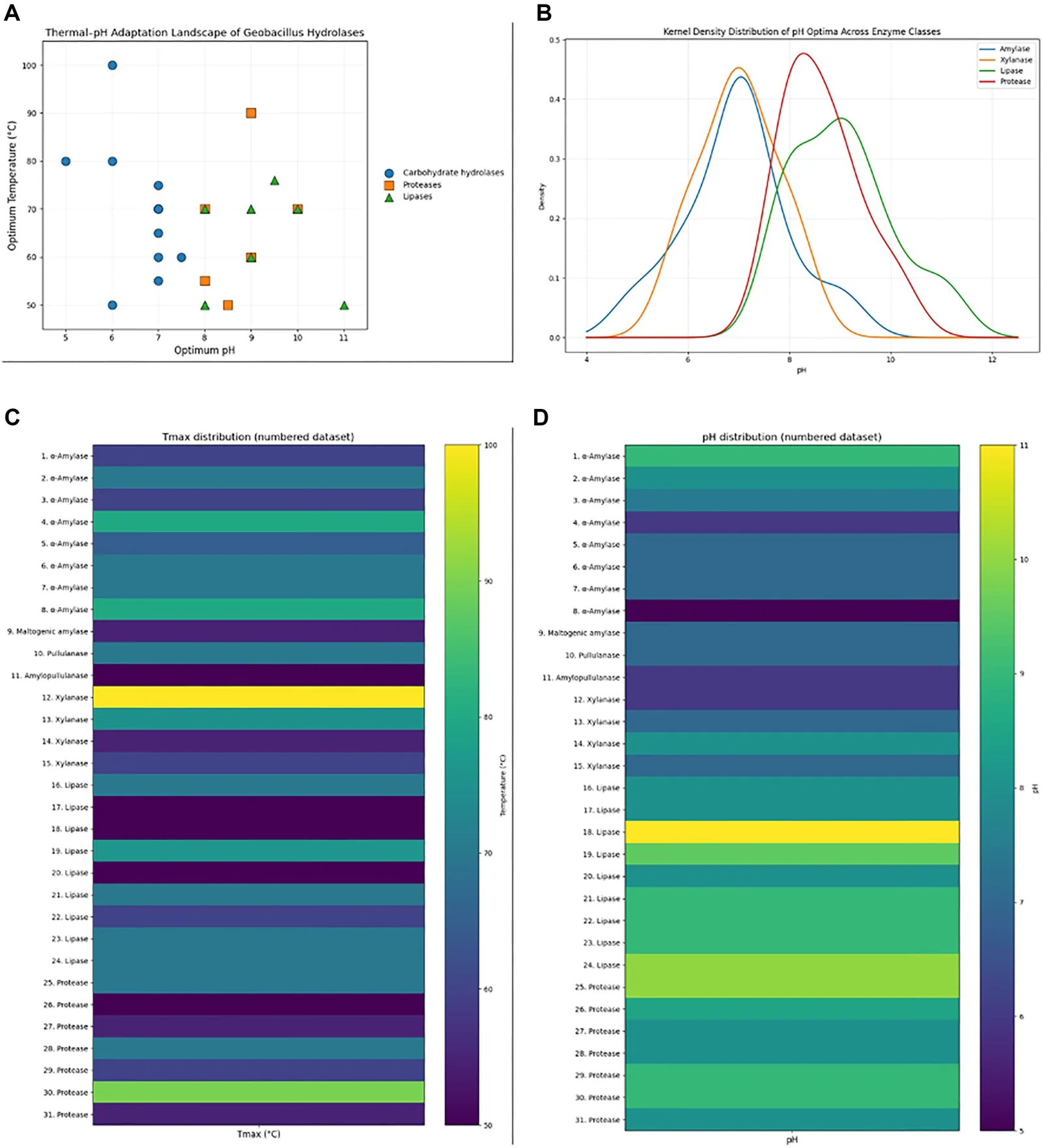
Comparative visualization of reported temperature and pH optima of *Geobacillus*-derived hydrolases compiled from the dataset presented in Table 4. (A) Thermal–pH landscape showing the relationship between reported optimum temperature (*T*_max_) and optimum pH (pH_max_) for carbohydrate hydrolases, proteases, and lipases. (B) Kernel density distributions of pH_max_ values for the enzyme categories represented in the dataset. (C) Heatmap of *T*_max_ values across the numbered 31-enzyme dataset. (D) Heatmap of pH_max_ values across the same dataset. All plotted values were derived directly from the data compiled in Table 4. Kruskal–Wallis analysis showed no significant difference in *T*_max_ among the major enzyme classes (*p* > 0.05), whereas pH_max_ distributions differed significantly among classes (*p* < 0.01).

The comparative *T*_max_–pH_max_ synthesis presented here does not constitute a formal meta-analysis, as the underlying enzymatic assays were conducted under heterogeneous experimental conditions. However, rather than seeking absolute quantitative convergence, this review focuses on directional trends and clustering behavior across enzyme classes. Despite variability in assay design, pH optima remain comparatively robust indicators of catalytic preference, as they reflect intrinsic protonation requirements of active-site residues rather than external reaction parameters. Consequently, recurrent pH clustering within enzyme classes—observed across independent studies—may reflect underlying biochemical constraints rather than methodological artifacts.

Crude extract data were intentionally excluded in order to reduce variability associated with mixed enzyme systems and non-standardized protein concentrations (McDonald & Tipton, 2022). Only studies reporting defined purification procedures and measurable specific activity values were included. *T*_max_ and pHmax were selected as the primary comparative parameters because they are the most consistently reported catalytic descriptors in thermophilic enzyme studies and provide direct, experimentally determined indicators of catalytic preference under defined assay conditions (Herlet et al., 2017). Given substantial heterogeneity among studies such as differences in substrate specificity, buffer composition, reaction time, and potential protein engineering modifications, a formal inferential meta-analysis was not considered appropriate. Instead, descriptive statistics were used to identify observable trends. The present synthesis should therefore be interpreted as an exploratory aggregation of reported catalytic maxima rather than a statistically weighted meta-analysis. The compiled dataset comprised 31 enzymes, including 15 carbohydrate-active hydrolases, 7 proteases, and 9 lipases. A descriptive summary of the aggregated *T*_max_ and pHmax values is presented in Table 4.

As summarized in Table 4, *T*_max_ values across all classes predominantly cluster within the 60–75 °C range, despite the presence of occasional outliers. This relatively narrow thermophilic window is consistent with the ecological origin of *Geobacillus* species and with previously reported thermal activity profiles of thermophilic enzymes (Shomali & Danish-Daniel, 2024; Wang et al., 2025). Instances of *T*_max_ ≥ 90°C are observed only in isolated enzymes, suggesting that extreme hyperthermophilic activity maxima may represent enzyme-specific structural adaptations rather than a general genus-level characteristic (Gault et al., 2021).

In contrast, pH_max_ values reveal clearer class-level differentiation. Carbohydrate-active hydrolases are centered on near-neutral conditions (mean pH_max_ ≈ 7.0), consistent with many reported thermophilic glycosidases (Ghosh et al., 2020; Iacono et al., 2023). Proteases exhibit a moderate alkaline shift (mean pH_max_ ≈ 8.6), while lipases display the strongest alkaline displacement, with pH_max_ values extending up to 11. Such alkaliphilic tendencies are frequently associated with industrially relevant proteases and lipases operating under detergent-like or lipid-rich conditions (Cherif et al., 2011; Hasan et al., 2010; Saraçoğlu et al., 2013). To visualize the relationship between *T*_max_ and pHmax, the compiled values were projected onto a two-dimensional thermal–pH view (Figure 3A).

As summarized in Figure 3D, the thermophilic phenotype of Geobacillus enzymes is characterized by thermal convergence coupled with pH-axis diversification. This pattern is further reinforced by heatmap analyses (Figure 3C and D), which highlight temperature clustering alongside class-dependent pH differentiation. Together, these observations capture both conserved and variable catalytic features without requiring enzyme-specific evolutionary assumptions.

The projection reveals overlapping yet directionally distinct tendencies. Carbohydrate-active hydrolases cluster predominantly within a neutral–thermophilic region, typically between pH 6.0–7.5 and 60–75 °C. This comparatively narrow pH distribution may reflect mechanistic constraints associated with catalytic acid–base chemistry during glycosidic bond cleavage, where precise protonation states of active-site residues are required (Cheng & DeYonker, 2022).

The observed pattern, thermal convergence coupled with pH-axis divergence, suggests that thermophilic activity may define a shared thermal operating window, while functional diversification is preferentially expressed through pH adaptation. This separation of thermal robustness from pH specialization can be interpreted as reflecting common catalytic trends rather than strictly independent enzyme-level variation.

Proteases occupy a moderately alkaliphilic space (pH 8–10) and exhibit broader thermal dispersion. This variability may stem from known structural diversity between serine and metalloprotease families and from selective pressure favoring alkaline tolerance in applied settings (Kaur et al., 2020; Sharma et al., 2019; Ullah et al., 2022). Compared with carbohydrate-active enzymes, proteases appear less constrained along both axes.

Lipases show the most pronounced alkaline specialization, with several enzymes displaying pHmax values near or above pH 10. Thermal dispersion within this class is comparatively moderate. The stronger pH shift observed for lipases is often associated with structural features such as surface hydrophobicity and lid-domain dynamics that influence activity under alkaline conditions (Khan et al., 2017). Relative to proteases, lipases demonstrate a clearer directional displacement along the pH axis than along the temperature axis.

Taken together, two broader tendencies emerge. First, thermophilic catalytic maxima are generally observed within a similar range across all hydrolase classes, suggesting a shared thermophilic tendency. Second, differentiation among functional classes is more pronounced along the pH axis than along the temperature axis, indicating that pH adaptation may vary in a class-dependent manner within *Geobacillus* hydrolases. It should be emphasized that these observations reflect descriptive trends derived from heterogeneous literature sources. The landscape representation provides a visual overview of catalytic tendencies but does not, by itself, establish statistically supported separation among functional classes. Nonetheless, positioning reported *T*_max_ and pH_max_ values within a shared comparative space provides a clearer perspective on catalytic adaptation patterns and offers a structured basis for future integration of additional stability metrics, such as thermal half-life or residual activity, once more standardized data become available. To further evaluate class-level differences, a non-parametric Kruskal–Wallis test was applied because of unequal sample sizes and the absence of normality assumptions; the corresponding statistical outcomes are reported in Figure 3.

To further examine temperature-related trends, Tmax values were visualized using a heatmap representation (Figure 3C). The distribution reveals that temperature optima cluster within a relatively narrow thermophilic range across enzyme classes. In contrast to temperature, pH-related patterns reveal clearer class-dependent differentiation. Visualization of pH_max_ values using a heatmap (Figure 3D) shows that carbohydrate-active hydrolases are predominantly clustered around near-neutral conditions, whereas proteases and lipases display a consistent shift toward alkaline pH ranges. This separation is not absolute but remains directionally consistent across the dataset, indicating that pH specialization represents a key axis of functional differentiation among *Geobacillus*-derived enzymes. To illustrate this divergence more clearly, pHmax distributions were independently visualized using kernel density plots (Figure 3B). A descriptive summary of *T*_max_ and pH_max_ values across major classes of *Geobacillus*-derived hydrolases is provided in Table 7, supporting the comparative trends identified in this section. Lipases and proteases demonstrate a consistent alkaline displacement relative to carbohydrate-active hydrolases, reinforcing the notion that thermophilic catalytic optima are conserved at the genus level, whereas alkaliphilic specialization appears to be class-dependent.

**Table 7:** Descriptive summary of reported temperature of maximum activity (*T*_max_) and pH of maximum activity (pH_max_) across major classes of *Geobacillus*-derived hydrolases.

Class	*N*	Mean *T*_max_ (°C)	Median *T*_max_ (°C)	*T*_max_ (°C)	Mean pH_max_	pH range
Carbohydrate-active hydrolases	15	68	70	50–100	7.0	5.0–9.0
Proteases	7	64.3	60	50–90	8.6	8.0–10.0
Lipases	9	63	70	50–76	9.1	8.0–11.0

*Note*. Values represent aggregated data from purified or partially purified enzymes extracted from peer-reviewed studies.

## Biotechnological applications

### Food industry applications

Enzymes from *Geobacillus* species have diverse applications in the food industry due to their thermostability, which enhances processing efficiency, improves product quality, extends shelf life, and reduces contamination risks (Khaswal et al., 2022; Salvador et al., 2025).

Amylases, which hydrolyze starch, are among the most widely used enzymes in this sector. *Geobacillus* amylases reduce dough viscosity in breadmaking, improving volume, texture, taste, color, and shelf life while delaying staling (Salvador et al., 2025). Amylase from *G. thermoleovorans* NP54 produces maltotetrose syrups (sweeteners) via starch saccharification (Novik et al., 2019). In addition, thermostable amylases reduce turbidity and viscosity during fruit juice clarification and facilitate starch gelatinization during mashing processes, thereby enhancing extraction efficiency.

Proteases that hydrolyze proteins are key in dairy, bakery, and food processing. Metalloendopeptidases like thermolysin from *Geobacillus* species are used in the synthesis of bioactive peptide derivatives and protein hydrolysates (Hussein et al., 2015; Najar & Thakur, 2020). For example, serine protease from *G. stearothermophilus* CAU209 hydrolyzes whey protein for antihypertensive hydrolysates (Chang et al., 2021).

Lipases that hydrolyze fats aid in the production of flavor compounds, improvement of lipid digestibility, and conversion of fats into high-value products. Recombinant lipase from *G. stearothermophilus* L1 hydrolyzes solid lipids like palm oil and beef tallow substrates that are typically resistant to mesophilic lipases. Xylanases can facilitate fruit-juice processing through the degradation of hemicellulosic components, and the application of a thermostable xylanase from *G. vulcani* GS90 has been demonstrated in fruit-juice enrichment (Algan et al., 2021). Xylanases are also relevant to pulp and paper processing, where hemicellulose degradation can facilitate subsequent processing and reduce chemical requirements (Phuyal et al., 2023).

### Bioenergy production

Biomass from plant and animal materials, such as agricultural waste, can be converted into solid, liquid, or gaseous bioenergy through physical, chemical, and biological processes. This renewable source offers low CO₂ emissions and sulfur content. Thermostable enzymes from *Geobacillus* species enhance production efficiency, particularly at high temperatures. These species have been widely reported in lignocellulosic biomass conversion to biofuels, fermenting both pentose and hexose sugars while producing key enzymes such as cellulases (Hussein et al., 2015), xylanases (Bhalla et al., 2022), α-amylases, and glycoside hydrolases (Baykara et al., 2021). These break down biomass into fermentable sugars for bioethanol and other fuels, positioning *Geobacillus* as promising for second-generation biofuels. In addition, lipases derived from the genus catalyze transesterification reactions, enabling biodiesel synthesis from lipid-rich feedstocks. The high-temperature tolerance of these enzymes can improve reaction rates and reduce contamination risks, highlighting the potential relevance of Geobacillus-derived enzymes for sustainable bioenergy (Khaswal et al., 2022; Soy et al., 2021).

### Detergent industry

Enzymes from *Geobacillus* species are widely used in detergents due to their stability under high temperatures, alkaline pH, and chemicals, improving cleaning efficiency, reducing energy consumption, and enabling eco-friendly products (Khaswal et al., 2022). Proteases, among the most common, break down protein-based stains. Examples include a thermostable protease from *G. stearothermophilus* B78 (Bayoumi et al., 2007), a serine protease from *Geobacillus* sp. GS53 stable against surfactants and organic solvents (Baykara et al., 2021), and a novel serine protease from *G. toebii* LBT tolerant to haloalkanes, solvents, and SDS (Thebti et al., 2016).

Lipases effectively remove oil and grease stains (Guncheva & Zhiryakova, 2011; Khaswal et al., 2022). A thermoalkaliphilic lipase from *G. stearothermophilus* FMR12 supports commercial detergent formulations by removing fabric stains (Abol Fotouh et al., 2016). Amylases target starch-based stains and are often combined with proteases in commercial formulations. The intrinsic thermal and alkaline stability of these enzymes supports washing performance under harsh domestic and industrial laundering conditions (Khaswal et al., 2022).

## Challenges and future directions in thermostable biocatalysis from *Geobacillus*

Research on *Geobacillus*-derived enzymes has generated an extensive body of knowledge, yet much of it remains enzyme-specific and fragmented. Individual proteases, lipases, and glycosidases have been characterized in detail, but systematic integration across structural determinants, catalytic behavior, and ecological adaptation remains limited. As discussed throughout this review, thermostability in *Geobacillus* can be interpreted as reflecting recurring structural and functional tendencies across enzyme classes rather than a collection of unrelated enzymatic traits. Translating this conceptual view into experimentally measurable and comparable parameters, however, remains challenging.

Methodological heterogeneity continues to constrain comparative synthesis. Although optimum temperature and pH values are routinely reported, complementary stability descriptors—such as thermal half-life, residual activity profiles, and unfolding thermodynamics—are inconsistently documented. Variations in substrates, assay conditions, and purification strategies further complicate cross-study interpretation. For comparative analyses, these inconsistencies reduce the strength of comparative inference and highlight the need for more standardized reporting practices in thermophilic enzyme characterization (Rigoldi et al., 2018; Vieille & Zeikus, 2001).

Bridging structural insight with predictive capability represents another key challenge. While hydrophobic core packing, salt-bridge networks, and controlled rigidity are widely associated with thermostability, their quantitative contributions to catalytic robustness across multiple *Geobacillus* enzymes remain insufficiently resolved. Integrative approaches combining structural bioinformatics, comparative genomics, and dynamic modeling may help clarify whether thermostable behavior can be anticipated prior to extensive experimental screening.

The catalytic landscape presented in this review suggests that thermophilic temperature optima are broadly conserved across hydrolase classes, whereas differentiation along the pH axis appears more function-dependent. Understanding how these adaptive dimensions co-evolve—and whether they emerge from shared or class-specific structural constraints—remains an important area for future research (Vieille & Zeikus, 2001).

From an applied perspective, translation into industrial systems still lags behind laboratory characterization. Long-term operational stability, tolerance to process fluctuations, and performance under realistic reactor conditions are not yet systematically evaluated for many *Geobacillus* enzymes. Integrating biochemical characterization with process-level validation will therefore be essential for moving beyond proof-of-concept applications. Ultimately, advancing thermostable biocatalysis in *Geobacillus* will depend on coordinated efforts that integrate structural biology, standardized enzymology, comparative analysis, and scalable bioprocess evaluation. Treating thermostability as a feature that can be increasingly understood and potentially predicted through integrated analysis may open new avenues for rational enzyme discovery and engineering within thermophilic systems.

## Conclusion

Overall, the comparative analysis indicates that *Geobacillus*-derived hydrolases operate within a broadly shared thermophilic temperature range, whereas their pH optima show clearer enzyme-class-dependent differentiation.

Common stabilizing features—such as strengthened hydrophobic cores, coordinated electrostatic networks, and a carefully balanced rigidity–flexibility profile—recur across different enzyme types, highlighting recurring stabilization features across enzyme classes. By linking these structural characteristics to comparative catalytic patterns, this review provides an integrative perspective that complements enzyme-specific descriptions. Moving forward, the development of standardized evaluation criteria and closer integration of structural analysis with functional assessment will be critical for transforming these comparative insights into predictive and application-driven strategies for thermostable biocatalyst design.

These findings provide a structured basis for future research on thermophilic enzymes, particularly in linking structural determinants with catalytic performance across enzyme classes. While the patterns identified here offer useful guidance for enzyme discovery and engineering, further experimental validation and standardized comparative datasets will be necessary to support more predictive approaches.

## Data Availability

No data available.
